# Genetic polymorphisms and epigenetic regulation of survivin encoding gene, *BIRC5*, in multiple sclerosis patients

**DOI:** 10.1186/s12865-019-0312-1

**Published:** 2019-08-22

**Authors:** Dariush Rahban, Forogh Mohammadi, Mehdi Alidadi, Taha Ghantabpour, Pedram Abbasi Ghasem Kheyli, Majid Ahmadi

**Affiliations:** 1 Department of Nanomedicine, School of Advanced Medical Technologies, Tehran University of Medical Sciences, Tehran, Iran; 2 Agriculture faculty, Department of Veterinary, Kermanshah Branch, Islamic Azad University, Kermanshah, Iran; 3 Department of Anatomy, School of Medicine, Tehran University of Medical Sciences, Tehran, Iran; 4 Paramedical Department, Shiraz University of Medical Sciences, Shiraz, Iran; 5 Stem Cell Research Center, Tabriz University of Medical Sciences, Daneshghah St., Imam Reza Hospital, Tabriz, Iran; 6 Student’s Research Committee, Tabriz University of Medical Sciences, Tabriz, Iran

**Keywords:** Multiple sclerosis, *BIRC5* gene, Survivin, Apoptosis, microRNA

## Abstract

**Background:**

The persistent the inflammatory condition in multiple sclerosis (MS) may due to the aberrant regulation of the elimination of the pathogenic autoreactive lymphocytes through apoptosis. Survivin, encoded by the BIRC5 gene, has been indicated to be involved in the regulation of apoptosis. This survey intended to investigate the genetic and microRNA mediated regulation of survivin in relapsing-remitting MS (RRMS) disease.

**Results:**

It was observed that the C allele (OR = 1.38, 95% CI = 1.05–1.348, *P* = 0.022) and CC genotype (OR = 1.84, 95% CI = 1.06–3.19; *P* = 0.029) in the rs9904341 polymorphism increased the disease risk. Furthermore, miR-34a was significantly downregulated (Fold change = 0.41, *P* = 0.001) in the PBMCs from RRMS subjects. Survivin mRNA expression in PBMCs and serum survivin level were increased in RRMS patients in comparison to the controls. Downregulation of miR-34a was negatively correlated with increased survivin level.

**Conclusion:**

Although the genetic polymorphism of *BIRC5* gene was associated with the disease risk, miR-34a was suggested to be involved in the regulation of survivin in the RRMS patients.

## Background

Multiple sclerosis (MS) is considered as a complex, multifactorial, and demyelinating disease that is related to a damage to the axonal myelin sheaths and is accompanied by neuronal degeneration. During MS, there is a loss in oligodendrocyte alongside with axonal damage, blood-brain barrier (BBB) leakage, and finally recruitment of immune cells that finally leads to central nervous system (CNS) inflammation [1]. MS is markedly prevalent among young adults, in which the mean age of the patients range from 20 to 45 [2]. MS is typically classified into four main groups according to the disease duration, including relapsing remitting MS (RRMS), secondary progressive MS (SPMS), primary progressive MS (PPMS), and progressive relapsing MS (PRMS) [3]. MS oftentimes represent a relapsing and remitting picture in its early stages, and as the disease course advances, symptoms are intensified for a while and then an amelioration occurs, which is named as RRMS, being reported in 80–85% of subjects [4, 5]. The exact etiology and pathogenesis of MS is not clear; nonetheless, a bulk of surveys indicate that the disease is the result of interactions between the immune system, inflammatory and apoptotic responses, which might be occurred at the periphery or the CNS [6]. Results from microarray studies conducted on the peripheral blood mononuclear cells (PBMC) obtained from MS patients in the relapsing phase indicated suppression of cell death through impaired apoptosis mechanisms [7].

*Baculoviral IAP repeat containing 5* (*BIRC5*) gene encodes a 16.5 kDa protein called survivin, which belongs to inhibitor of apoptosis gene family (IAP) and is involved in the apoptosis and cell proliferation process [8]. Survivin has been reported to regulate the immune system, in which the development and differentiation of effector CD4+ T cells, hemostasis of CD8+ memory T cells, as well as the proliferation of activated T cells are controlled by survivin [9].

It has been shown that single nucleotide polymorphisms (SNPs) located in the promoter of the BIRC5 gene might be involve in the alteration of expression level of survivin [10]. The rs9904341 SNP is found in regions encoding for the cell cycle dependent elements as well as the repressor binding site of the cell cycle homology regions and was associated with increased mRNA and protein expressions of survivin in cancers [11, 12]. Furthermore, rs17878467 and rs8073069 SNPs in the *BIRC5* promoter region have been reported to modulate the expression of survivin. While rs17878467 increased *BIRC5* promoter activity in HeLa cell lines [12], rs8073069 was associated with overexpression of survivin in the esophageal cancer [13].

microRNAs (miRNAs) are short and single stranded, non-coding RNA molecules composed of approximately 22 nucleotides that are involved in controlling the gene expression. It has been reported that almost 60% of human genes are attributed to at least one miRNA binding site. miRNAs primarily suppress the translation of target genes and eventuate in downregulation of genes. During the biogenesis of miRNAs, RNA polymerase II transcribes the gene encoding miRNA to a long RNA strand named as primary miRNAs (pri-miRNAs). Then Drosha, an endonuclease, catalyzes the development of pri-miRNA to precursor miRNAs (pre-miRNAs). Aftermath, the exportin-5 mediated the transportation of pre-miRNA to cytoplasm, where it is cleaved to a double strand miRNA by Dicer. The active miRNA strand in the ribonucleoprotein complex, namely RNA-induced silencing complexes (RISCs) binds to 3′-untranslated region (UTR) of a mRNA, culminating in gene repression [14].

On the one hand, it has been reported that survivin was highly expressed in PPMS patients [15]. As well, an overexpression of survivin in T cells from active MS patients compared with stable MS patients has been reported that correlated with cellular resistance to apoptosis as well as with disease activity manifestations, including the number of enhanced lesions and disease duration [16]. On the other hand, numerous studies have indicated a dysregulated miRNA in various cell types in MS patients [17] and testified the involvement of genetic contribution in the etiology of this disease [18]. Considering these facts, this study aimed to evaluate the role of genetic implications (rs9904341, rs17878467, and rs8073069 SNPs in *BIRC5* gene) in the regulation survivin level. Additionally, important previously confirmed miRNAs (miR-16, miR-34a, miR-150, and miR-203a) in targeting the survivin mRNA was surveyed in the PBMCs of MS patients and investigated their involvement in the regulation of survivin mRNA expression in PBMCs as well as survivin serum concentration.

## Methods

### Patients and control subjects

This case-control study was carried out on 200 RRMS patients referred to Fars Multiple Sclerosis Society and 200 healthy controls. Patients who had chronic inflammatory disorders, cancer, autoimmune disease, drug intake etc. were excluded. All the patients were in the relapsing state. Furthermore, healthy controls had no autoimmune disease in themselves as well as their family members. Individuals in the case and control groups were age- and sex- matched. Patients were diagnosed as having MS based on the modified McDonald criteria [19, 20] and the disability score of patients was determined by Expanded Disability Status Scale (EDSS) [21]. In this study, MS subjects were selected from those in the remitting state that had received no immunomodulating medications for at least 3 months before sampling. The protocol of this study was approved by the Human Research Ethics Committee from the Shiraz University of Medical Sciences, Fars, Iran and written informed consent forms was taken by all subjects. Blood samples from MS patients were collected when the disease was clinically diagnosed. To perform experiments, 10 ml of venus blood from all participants was collected in EDTA tubes via venipuncture.

### Real-time PCR genotyping of SNPs

Using Real-time allelic discrimination Taq-Man assays (Applied Biosystems, Foster City, USA), patients and controls were genotyped for rs9904341, rs17878467, and rs8073069 SNPs in *BIRC5* gene. The reactions mixture contained 5 μl of genomic DNA (200 ng/μl), 5 μl of Taq-Man Master Mix (Applied Biosystems, Foster City, USA), 0.5 μl of Taq-Man Genotyping Assay mix containing FAM or VIC labeled probes and primers (Applied Biosystems, Foster City, USA), and H_2_O to reach a final volume of 25 μl. Real-Time allelic discrimination PCR condition that was performed via StepOnePlus Real-Time PCR system (Applied Biosystems, Foster City, USA) was: initially 60 °C for 30 s and then 95 °C for 10 min, and then 40 amplification cycles in 95 °C for 15 s and 60 °C for 1 min, and ultimately 60 °C for 30 s.

### PBMC separation, RNA extraction, cDNA synthesis

To attain PBMCs from 50 RRMS patients and 50 healthy individuals, the Ficoll-Hypaque density gradient approach was done. To isolate the RNA, the MiRNeasy Mini kit (Qiagen, Germany) was used. For determination of the yield and purity of isolated RNAs, a Nano Drop spectrophotometer at 260/280 nm (Nano Drop ND-2000C Spectrophotometer, Thermo Fisher Scientific, USA) was used. The first strand complementary DNA (cDNA) was synthesized using the miScript II RT Kit (Qiagen, Germany) based on the manufacturer’s protocol.

### Real-time PCR quantification of miRNA and survivin expression

Measurement of the miRNA expression levels (including miR-16, miR-34a, miR-150, and miR-203a) was carried out by miScript SYBR Green PCR Kit (Qiagen, Germany) and StepOne Plus Real-Time PCR (Applied Biosystems, Foster City, CA, USA). Each reaction mixture contained 6 μl cDNA template, 10 μl of SYBR Green PCR Master mix, 2 μl primers each, and RNase free water to a total volume of 25 μl. The Real-time quantitative PCR conditions were: initial 95 °C for 10 min, 40 cycles of 95 °C for 15 s, and 55 °C for 30 s, and finally 65 °C for 30 s.

Real-time PCR quantification of survivin mRNA level in PBMCs from RRMS patients and healthy controls was performed using RealQ Plus Master Mix Green High ROX (AMPLIQON, Denmark) and StepOne Plus Real-time PCR machine (Applied Biosystems, Foster City, CA, USA). The components of the mixture were 10 μl SYBR Green Master Mix, 8 μl cDNA, 0.4 μl primers each (forward primer of 5′-CCACCGCATCTCTACATTCA-3′ and reverse primer of 5′-GTCTGGCTCGTTCTCAGTGG-3′; adopted from Primer Bank; https://pga.mgh.harvard.edu/primerbank/), and RNase free H_2_O for a final volume of 25 μl. The thermocyclic conditions of Real-time PCR were: the holding step of 95 °C for 15 min, 50 cycles of 95 °C for 15 s, and 63 °C for 30 s, and then 70 °C for 1 min.

Comparative C_T_ method was exploited to calculate relative miRNA and survivin expressions as previously described by Livak and Schmittgen [22]. For normalizing the expression levels of target genes, the transcript levels of RUN6 (for miRNAs) and GAPDH (for survivin), as the housekeeping genes, were determined.

### Survivin concentration

To determine the survivin level, the serum samples were isolated from the peripheral blood of 50 patients and 50 control subjects. Survivin level was determined using enzyme-linked immunosorbent assay (ELISA) and a commercial kit (Human Survivin ELISA Kit, OriGene Technologies, Inc., Rockville, MD, USA).

### Statistical analysis

Genotype and allelic distribution between case and control groups were implemented by Chi-Square test. Pearson’s χ2-tests were applied to test for significance differences of both genotype and allele frequencies between two groups. The odds ratio (OR) and 95% confidence interval (CI) were calculated. The genotype distributions of chosen SNPs were tested for deviation from Hardy-Weinberg equilibrium in case and control. Determination of normality of scale data distribution was conducted using the Kolmogorov-Smirnov test. The independent *t*-test or ANOVA was used to compare the groups. The GraphPad Prism v. 6.00 (GraphPad Software, Inc., San Diego, CA, USA, www.graphpad.com) was exploited for plotting. SPSS software v. 21 (SPSS, Chicago, IL, USA) was used for data analysis. Data were presented as number and percentage or mean ± standard deviation (SD) with statistical significance at *P* < 0.05.

## Results

### Baseline characteristic of the study population

RRMS patients and healthy controls had the mean age of 43.60 ± 11.33 and 41.54 ± 15.74, respectively. The RRMS group and healthy control group were comprised of female/male ratio of 144 (72%)/56 (28%) and 145 (72.5%)/55 (27.5%). Therefore, the patients and controls were age- and sex-matched. In the patient group, the age of onset of RRMS was 27.65 ± 10.88 and duration of the diseases was 7.41 ± 3.15. RRMS patients demonstrated to have EDSS score of 3.89 ± 1.45 (Table 1).

**Table 1:** Baseline characteristics of the study participants

Characteristic	RRMS Patients (*n* = 200)	Healthy Controls (n = 200)
Female/Male	144 (72%)/56 (28%)	145 (72.5%)/55 (27.5%)
Age	43.60 ± 11.33	41.54 ± 15.74
Onset age	27.65 ± 10.88	–
Duration of the disease	7.41 ± 3.15	–
EDSS	3.89 ± 1.45	

*RRMS* Relapsing Remitting Multiple Sclerosis, *EDSS* Expanded Disability Status Scale

### Genotyping findings

According to the Table 1, the distribution of the genotypes in all three SNPs (rs9904341, rs17878467, and rs8073069) in the control group disclosed to have no significant distortion (*P* = 0.28, 0.21, and 0.52, respectively) from the Hardy–Weinberg equilibrium.

Among the genotyped SNPs, only rs9904341 demonstrated statistically significant difference with respect to the frequency of alleles and genotypes between the case and control groups. For this SNP, the major G allele was considered as the reference allele and the C allele was the minor allele, which was significantly prevalent in the patient group (Table 2). It was observed that the C allele had 47% frequency in the patient group and 39% in the control group. This allele significantly increased the risk of MS (OR = 1.38, 95% CI = 1.05–1.348, *P* = 0.022). on the other side, the GG genotype was the reference genotype. The CC genotype was detected in 24.5% of RRMS patients and 17% of the healthy subjects, and significantly increased the risk of the disease (OR = 1.84, 95% CI = 1.06–3.19; *P* = 0.029). However, the CG genotype had no significant difference between patient and control groups.

**Table 2:** The allele and genotype frequencies of the genotyped SNPs in the *BIRC5* gene in MS patients and healthy individuals (HWE value is a probability)

SNP	Alleles/Genotypes	MS Patients (*n* = 200) N (%)	Controls (*n* = 200) N (%)	*P v*alue	OR (95%CI)
rs9904341	C	188 (47)	156 (39)	**0.022**	1.38 (1.05–1.84)
	G	212 (53)	244 (61)	Reference	
	CC	49 (24.5)	34 (17)	**0.029**	1.84 (1.06–3.19)
	CG	90 (45)	88 (44)	0.238	1.31 (0.83–2.04)
	GG	61 (30.5)	78 (39)	Reference	
HWE			0.287		
rs17878467	T	68 (17)	73 (18.25)	0.642	0.91 (0.63–1.32)
	C	332 (83)	327 (81.75)	Reference	
	TT	9 (4.5)	4 (2)	0.242	2.05 (0.62–6.80)
	CT	50 (25)	65 (32.5)	0.110	0.70 (0.45–1.08)
	CC	144 (72)	131 (65.5)	Reference	
HWE			0.21		
rs8073069	C	163 (40.75)	150 (37.5)	0.346	1.15 (0.86–1.52)
	G	237 (59.25)	250 (62.5)	Reference	
	CC	35 (17.5)	26 (13)	0.249	1.42 (0.78–2.59)
	GC	93 (46.5)	98 (49)	0.993	1.00 (0.65–1.54)
	GG	72 (36)	76 (38)	Reference	
HWE			0.52		

*OR* odds ratio, *CI* confidence interval, *HWE* Hardy-Weinberg equilibrium

Values in bold shows significant *P* values

### Expression level of miRNAs

**Fig. 1:**
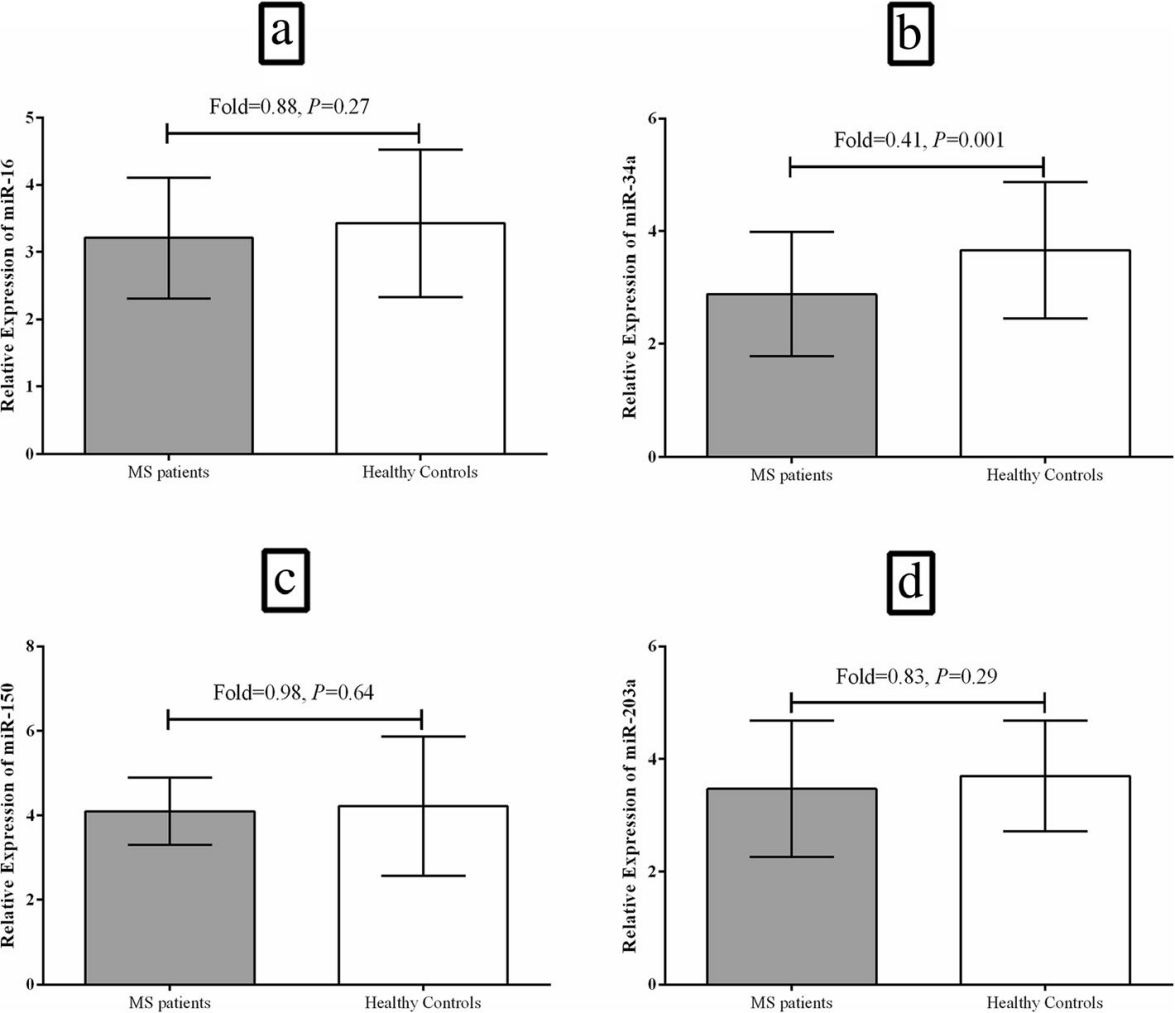
Representation of the expression of **a**; miR-16, **b**; miR-34a, **c**; miR-150, and **d**; miR-203a in the PBMCs from RRMS patients and the healthy subjects

**Fig. 2:**
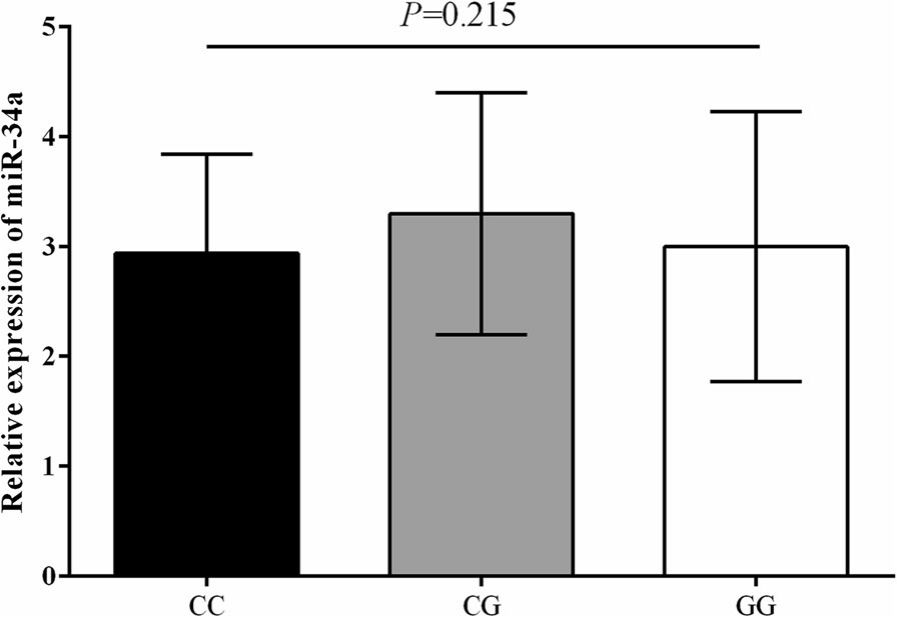
Bar graphs illustrates the relative miR-34a expression in the PBMCs from RRMS patients with the three genotypes of CC, CG, and GG for the rs9904341 SNP that had significantly different genotype frequencies between patients and controls

Among the evaluated miRNAs, only miR-34a indicated statistically significant difference in expression inside the PBMCs between the patients and controls. This miRNA was significantly downregulated in the PBMCs of the RRMS patients in comparison to the controls (Fold change = 0.41, *P* = 0.001, Fig. 1.b). Other miRNAs, including miR-16 (Fold change = 0.88, *P* = 0.27, Fig. 1.a), miR-150 (Fold change = 0.98, *P* = 0.64, Fig. 1.c), and miR-203a (Fold change = 0.83, *P* = 0.29, Fig. 1.d) were downregulated in the PBMCs of RRMS patients in comparison to healthy controls, but the differences were not statistically different. It was revealed that miR-34a transcript level had no statistically significant difference (*P* = 0.215) in the PBMCs from RRMS patients with the three genotypes of CC, CG, and GG for the rs9904341 SNP that had significantly different genotype frequencies between patients and controls (Fig. 2).

### mRNA expression and serum concentration of survivin

**Fig. 3:**
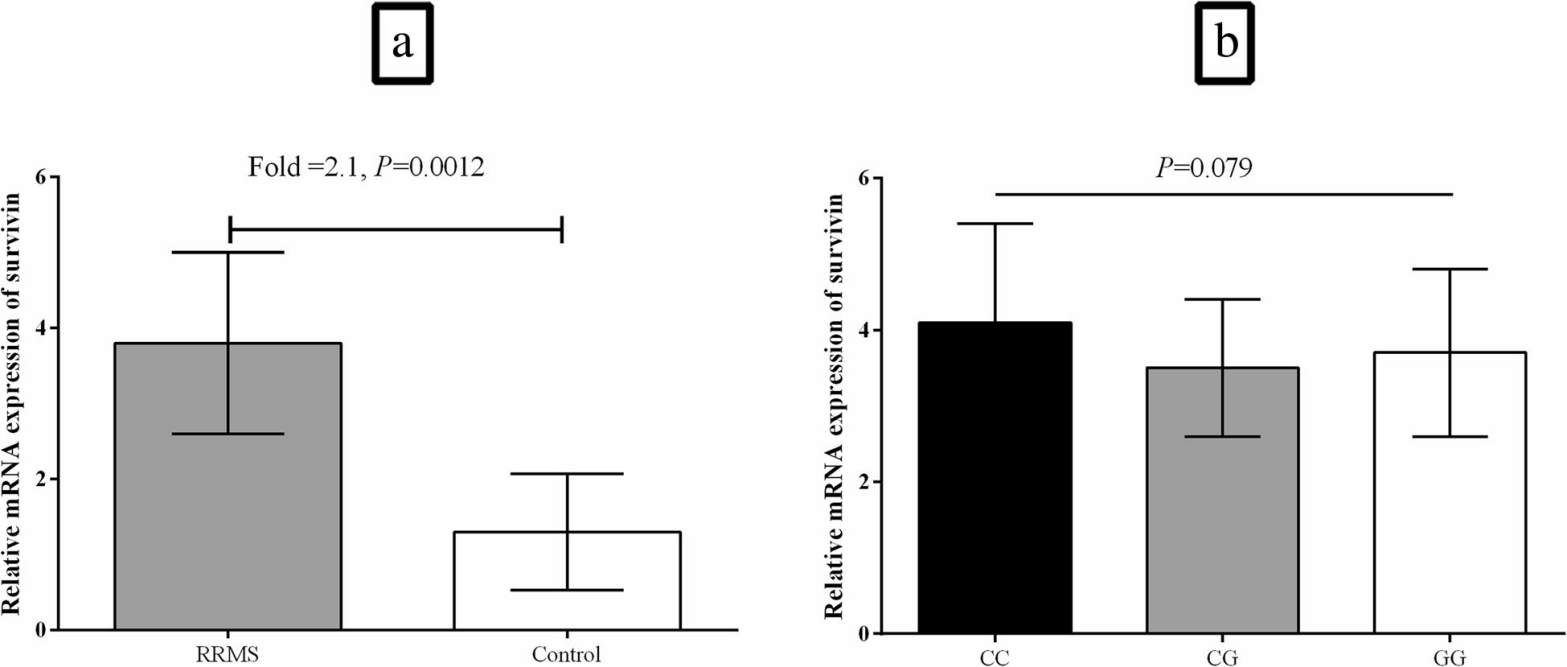
Bar graphs illustrates the relative mRNA expression of survivin in the PBMCs from RRMS patients in comparison to healthy controls (**a**) and in the RRMS patients with the three genotypes of CC, CG, and GG for the rs9904341 SNP (**b**) that had significantly different genotype frequencies between patients and controls

It was observed that mRNA expression of survivin was upregulated significantly in the PBMCs from RRMS patients compared with healthy control group (Fold change = 2.1, *P* = 0.0012; Fig. 3.a). Moreover, there was no statistically significant difference (*P* = 0.079) in the mRNA expression of survivin in the PBMCs from RRMS patients with the three genotypes of CC, CG, and GG for the rs9904341 SNP that had significantly different genotype frequencies between patients and controls (Fig. 3.b).

**Fig. 4:**
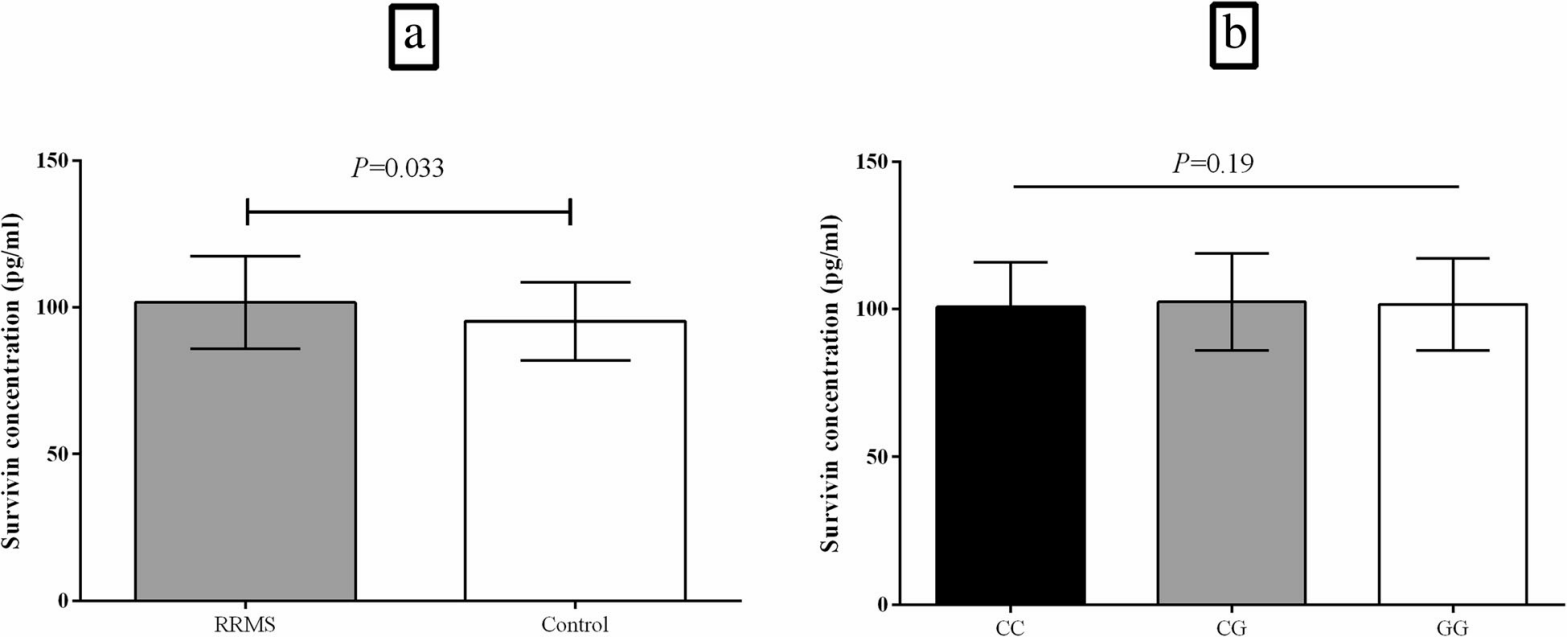
Bar graphs illustrates the survivin serum concentration in RRMS patients in comparison to healthy controls (**a**) and in the RRMS patients with the three genotypes of CC, CG, and GG for the rs9904341 SNP (**b**) that had significantly different genotype frequencies between patients and controls

The serum level of survivin was increased significantly in the RRMS patients compared with healthy subjects (101.66 ± 15.68 pg/ml vs. 95.27 ± 13.28 pg/ml, *P* = 0.033; Fig. 4.a). However, the serum level of survivin had no statistically significant difference (*P* = 0.19) among the RRMS patients with the three genotypes of CC (100.78 ± 15.14 pg/ml), CG (102.49 ± 16.43 pg/ml), and GG (101.63 ± 15.52 pg/ml) for the rs9904341 SNP (Fig. 4.b).

### Correlation analyses

Among the miRNAs, downregulation of the miR-34a demonstrated statistically significant negative correlation with the mRNA expression of survivin in PBMCs (*r* = − 0.23, *P* = 0.0012) as well as with the serum level of survivin (*r* = − 0.29, *P* = 0.0027). However, the correlation analysis indicated no relation between the expression of miRNAs and the EDSS score of RRMS patients.

mRNA expression of survivin in PBMCs as well as serum survivin level did not show statistically significant correlation with the EDSS score in RRMS patients (*r* = 0.29, *P* = 0.088 and *r* = 0.41, *P* = 0.37, respectively). Furthermore, the EDSS score did not have statistically significant difference among the RRMS patients with the three genotypes of rs9904341 SNP.

## Discussion

This study intended to survey the role of genetic variations (rs9904341, rs17878467, and rs8073069 SNPs located in *BIRC5* gene) as well as important miRNAs (miR-16, miR-34a, miR-150, and miR-203a) in the regulation of survivin. It was observed that both genetic variations and miRNA dysregulation might be involved in the disease pathogenesis. However, only miR-34a was shown to regulate the survivin mRNA expression and survivin serum level in RRMS patients.

Survivin play a role in the inhibition of apoptosis and is involved in the regulation of both cell proliferation and survival [8]. During malignancies and immune tolerance failure in the autoimmune settings, mitosis and resistance to apoptosis are involved in the pathogenesis of the disease. It has been reported that upregulation of survivin in various tumor cells eventuates in the tumor progression, resistance to drug, and decreased survival [9, 23]. Survivin modulates important mechanisms of the immune system, including promoted proliferation of T cells upon activation, differentiation of CD4^+^ T cells, and hemostasis of CD8^+^ memory T cells. Upregulation of survivin has been associated with increased expression of the vital molecules during antigen presentation to T cells and required co-stimulatory molecules, including major histocompatibility complex (MHC) class II and CD80/86 molecules [9]. With respect to such critical roles of survivin in the context of immune system, survivin has been investigated in the autoimmunity including, MS [9]. That notwithstanding, there is a paucity of understandings regarding the regulatory mechanism of survivin in the pathogenesis of autoimmune diseases.

It has been reported that removal of autoreactive lymphocytes, which might be regulated by apoptosis through the regulation of survivin, is aberrantly modulated in MS [24–26]. It was reported that survivin level was overexpressed in the resting T cells from PPMS patients [15]. Moreover, decreased expression of survivin level was disclosed in the peripheral T cells stimulated by interferon-β-1a (IFN-β-1a) ex vivo, inducing the apoptosis in the T cells [27]. Ex vivo overexpression of survivin induced T cells from patients with active MS [16]. Upregulated expression of survivin was detected in the mitogen stimulated resting lymphocytes isolated from RRMS patients [28]. It seems that survivin increases the survival and proliferation of autoreactive T cells in MS patients. In the current study, the serum level of survivin was higher that control subjects. Moreover, mRNA expression of survivin was upregulated in the PBMCs (that contain predominantly the lymphocytes) from RRMS patients.

Genetic polymorphisms of *BIRC5* gene has been associated with the modulation of expression level of survivin [10]. For example, the rs9904341 SNP was associated with increased mRNA and protein expressions of survivin in cancers [11, 12]. The rs17878467 SNP was associated with increased *BIRC5* promoter activity in HeLa cell lines [12], and the rs8073069 SNP in the *BIRC5* gene was associated with overexpression of survivin in esophageal cancer [13]. These polymorphisms impressed the response to treatment and the disease activity in rheumatoid arthritis patients [29]. This study for the first time evaluated the genetic polymorphisms in MS patients and indicated that the C allele and CC genotype in the rs9904341 SNP increased the disease risk. Nonetheless, this polymorphism was associated with neither survivin level in serum nor EDSS score in the RRMS patients.

Survivin expression is under the regulation of miRNAs. In the current study, important miRNAs regulating the survivin expression was selected miRTarBase (http://mirtarbase.mbc.nctu.edu.tw/php/index.php), the experimentally validated microRNA-target interactions database (http://www.microrna.org/microrna/home.do), miRDB (http://mirdb.org/cgi-bin/search.cgi), and already confirmed miRNAs by the published works. To the best of our knowledge, this was the first investigation of the survivin regulation through miRNAs in multiple sclerosis. We observed that miR-34a was downregulated in the PBMCs from RRMS patients that was inversely correlated with overexpression of survivin in PBMCs from RRMS patients as well as with the increased survivin level in the serum samples. Nonetheless, decreased levels of miR-34a did not impress the disease activity based on EDSS score. It seems that miR-34a regulates the expression of survivin in PBMCs, regardless of its involvement in the clinical presentation of RRMS patients with respect to EDSS.

## Conclusions

In consideration of all the findings, this was the first study, to the best of our knowledge, that intended to search for regulatory mechanisms of survivin expression in the MS disease. The genetic variations (rs9904341, rs17878467, and rs8073069 SNPs located in *BIRC5* gene) was studied and was detected that the C allele and CC genotype in the rs9904341 SNP increased the disease risk. Furthermore, miR-34a was downregulated in the PBMCs from RRMS subjects that was negatively correlated with the mRNA expression of survivin in the PBMCs as well as with the survivin level in the serum. The findings here could be considered preliminary insight into the survivin regulator mechanisms in MS disease that still needs to be enrichened in the future works.

## Data Availability

The data that support the findings of this study are available on request from the corresponding author. The data are not publicly available due to privacy or ethical restrictions. All data generated or analyzed during this study are included in this published article.

## Abbreviations

BBB: Blood-brain barrier
BIRC5: Baculoviral IAP repeat containing 5
CI: Confidence interval
CNS: Central nervous system
EDSS: Expanded disability status scale
ELISA: Enzyme-linked immunosorbent assay
IFN-β-1a: Interferon-β-1a
MHC: Major histocompatibility complex
miRNAs: microRNAs
MS: Multiple sclerosis
OR: odds ratio
PBMC: peripheral blood mononuclear cells
PPMS: Primary progressive MS
pre-miRNAs: precursor miRNAs
pri-miRNAs: primary miRNAs
PRMS: Progressive relapsing MS
RISCs: RNA-induced silencing complexes
RRMS: Relapsing-remitting MS
SD: Standard deviation
SNPs: single nucleotide polymorphisms
SPMS: Secondary progressive MS
UTR: 3′-Untranslated region
